# Having an Eating Disorder and Still Being Able to Flourish? Examination of Pathological Symptoms and Well-Being as Two Continua of Mental Health in a Clinical Sample

**DOI:** 10.3389/fpsyg.2018.02145

**Published:** 2018-11-15

**Authors:** Jan Alexander de Vos, Mirjam Radstaak, Ernst T. Bohlmeijer, Gerben J. Westerhof

**Affiliations:** ^1^Psychology, Health, & Technology, Centre for eHealth and Wellbeing Research, University of Twente, Enschede, Netherlands; ^2^Stichting Human Concern, Centrum voor Eetstoornissen, Amsterdam, Netherlands

**Keywords:** eating disorders, anorexia nervosa, bulimia nervosa, binge eating disorder, well-being, psychological well-being, positive functioning

## Abstract

**Introduction:** Eating Disorders (EDs) are serious psychiatric disorders, impacting physical and psychosocial functioning, often with a chronic course and high mortality rates. The two continua model of mental health states that mental health is a complete state, that is, not merely the absence of mental illness, but also the presence of mental health. This model was studied among ED patients by examining the presence and correlates of well-being and psychopathology. In addition, the levels of well-being were compared to the Dutch general population.

**Method:** A total of 468 female ED patients participated in this study during application and intake at a specialized ED treatment Center in the Netherlands. They filled out questionnaires about well-being (MHC-SF), general psychopathology (OQ-45), and ED psychopathology (EDE-Q). Categorical andmean well-being levels were calculated. Also, the relationships between these variables were examined with Pearson correlation and multiple hierarchical regression analysis.

**Results:** ED patients showed lower levels of emotional, psychological, and social well-being on average compared to the general population. About 26% of the ED patients experienced low levels of well-being (languishing). However, also 13% experienced high levels of well-being (flourishing), varying between 9% in Anorexia Nervosa to 25% in Binge Eating Disorder. ED psychopathology and general well-being showed a moderate negative correlation. For patients with Bulimia Nervosa and Binge Eating Disorder however no such correlation was found. Lower general psychopathology, not having a history of hospitalization for the ED, and adaptive personal functioning were correlated with well-being among ED patients.

**Conclusion:** This study shows initial support for the two continua model of mental health among ED patients. Psychopathology and well-being should be considered as related, but distinct dimensions of mental health in ED patients. Further research should focus on the possible reciprocal relationships between psychopathology and well-being during recovery. It is recommended to monitor well-being during treatment and to implement interventions for well-being to realize complete recovery for those patients with inadequate levels of well-being.

## Introduction

Eating Disorders (EDs) are serious psychiatric disorders (American Psychiatric Association, 2013). They often lead to severe psychological, physical, and social impairment and chronic conditions (Lowe et al., 2001; Jenkins et al., 2011; Mitchison et al., 2012; Mond et al., 2012). Anorexia Nervosa (AN) has the highest mortality rate of all psychiatric disorders, because of the severe physical conditions and suicide (Harris and Barraclough, 1998; Hoek, 2006). The lifetime prevalence estimates for women with eating disorders are 0.9% for AN, 1.5% for Bulimia Nervosa (BN), 3.5% for Binge Eating Disorder (BED), and 0.3, 0.5, and 2.0% among men (Hudson et al., 2008).

The severity and chronicity of ED disorders might explain the main focus in research on psychopathological symptoms and the ignorance of well-being (Tomba et al., 2014). However, mental health is more than the absence of mental illness or psychopathological symptoms. Mental health is also about the presence of well-being (Jahoda, 1958; Seligman and Csikszentmihalyi, 2000; Keyes, 2002, 2012; World Health Organization, 2005; Westerhof and Keyes, 2010). Well-being consists of three components, namely emotional, psychological, and social well-being (Ryff and Keyes, 1995; Keyes, 2002; Westerhof and Keyes, 2010). Emotional well-being is about satisfaction with life and positive affect (Diener et al., 1999; Lamers et al., 2011). Psychological well-being concerns optimal psychological functioning and consists of six dimensions: positive relationships, self-acceptance, environmental mastery, autonomy, personal growth, and purpose in life (Ryff, 1989; Ryff and Keyes, 1995). Social well-being is about optimal functioning in the societal context and consists of five dimensions: social contribution, integration, actualization, acceptance, and coherence (Keyes, 1998). People with high levels of well-being have been described as flourishers, whereas those who are low on well-being languishers (Keyes, 2002, 2005).

Studies among the general and clinical populations showed the importance of well-being in mental health. Psychopathology and well-being are not two opposites of one dimension, but represent two distinct, yet negatively related dimensions of mental health, the so-called two continua model (Keyes, 2005, 2006, 2007; Keyes et al., 2008; Lamers et al., 2011; Peter et al., 2011; Magalhães and Calheiros, 2017; Perugini et al., 2017; Franken et al., 2018). This means that someone with psychopathology may still have high levels of well-being, and that someone with low levels of well-being does not necessarily also have psychopathology. Further evidence for the two continua model comes from studies that show that pathological symptoms and well-being have different correlates. For example, Westerhof and Keyes (2010) found different correlates for psychopathology (age, married, employed, number of illnesses, subjective health) compared to well-being (gender, migration, subjective health) in the general population.

The two continuum structure of mental health in clinical samples is further substantiated by results of a randomized controlled study of Acceptance and Commitment Therapy, where 64% of the participants improved either on depressive symptoms, or on well-being, but not on both (Trompetter et al., 2017).

Well-being has been considered as an important component of recovery in psychological treatments (Fava, 1996) and specific therapies for improving well-being among clinical populations have been developed (Fava et al., 1998, 2005; Fava and Ruini, 2003; Gilbert, 2009; Bolier et al., 2013; Weiss et al., 2016). Even though persons who are recovered from an ED consider several aspects of well-being as fundamental criteria for ED recovery in addition to symptom remission (de Vos et al., 2017), the important role of well-being for mental health has been widely neglected in research among ED patients (Tomba et al., 2014). Some researchers have focused on health-related components among ED patients, such as quality of life or subjective well-being (de la Rie et al., 2005, 2007; Doll et al., 2005; Jenkins et al., 2011; Mond et al., 2012; Tomba et al., 2014). Two studies examined the presence of psychological well-being (PWB) among ED outpatients (Tomba et al., 2014, 2017). In the first study it was found that ED patients had impaired PWB compared to a healthy control group (Tomba et al., 2014). Furthermore, patients with Bulimia Nervosa had greater impairment on all psychological well-being scales compared to a control group, whereas patients with Binge Eating Disorder showed greater impairment only on autonomy, environmental mastery, and self-acceptance, and patients with AN only on positive relationships with others and self-acceptance. This study also found that impaired levels of PWB were independent from the presence of psychopathology, indicating that the presence of PWB does not simply correspond to the absence of psychopathology (Tomba et al., 2014). In the other study, change in PWB among ED patients during outpatient cognitive-behavioral-based treatment was examined. It was found that patients improved on the PWB dimensions during treatment (Tomba et al., 2017). However, after treatment, ED patients still showed impaired positive relationships with others and self-acceptance compared to controls (Tomba et al., 2017). Whereas, previous studies addressed several aspects of well-being, no study has examined all levels of well-being or the two continua model among ED patients.

Psychopathology and well-being might have different correlates among ED patients. Correlates related to the severity of ED psychopathology are, among others, personality traits, emotion regulation difficulties, psychiatric pathology such as depression and anxiety, traumatic past, and body mass index (BMI) (Johnson et al., 2002; Jacobi et al., 2004; Cassin and Von Ranson, 2005; Costa et al., 2008; Haynos et al., 2015). Correlates with well-being have not been examined yet, while this may provide information on which ED patients might be vulnerable to inadequate well-being, which would give guidance in the treatment of eating disorders.

In summary, there is growing support that well-being is an important dimension of mental health, while no studies have been conducted on all three levels of well-being. This study aimed to answer the question whether the two continua model for mental health can be confirmed among a clinical sample of ED patients by addressing the following research questions:

1. What are the levels of well-being and the proportions of ED patients who are languishing and flourishing, and does this differ from the general population?
2. To what extend are psychopathology and well-being related in ED patients?
3. What are the correlates of psychopathology and well-being in ED patients?

Question *one* and two are also examined per eating disorder type in addition to the overall sample of ED patients. For research question *one* we expect that ED patients will have lower levels of well-being and are less likely to flourish compared to the general population. However, we also expect substantial variation in well-being between ED patients, with a substantial part showing moderate to high well-being, as an indicator that well-being functions as a distinct continuum of mental health. Regarding the specific ED types, based on the study of Tomba et al. (2014), we expect more patients with BN languishing compared to other ED types and more patients with AN flourishing compared to other ED types. For research question *two* we expect a low to moderate negative correlation between (a) general psychopathology and well-being, and (b) eating disorder psychopathology and well-being as additional indicators for the two continua model. We expect no differences between ED types. For research question *three* we expect, based on the two-continua model, that different correlates will be associated with ED pathology and well-being. The analysis of correlates is however explorative since this is the first study to examine correlates for well-being in eating disorder patients. Overall, we expect that the two-continua model of complete mental health can be replicated among ED patients, and we consider the results of all three research questions as potential support for the two-continua model.

## Methods

### Participants and procedure

A cross-sectional research design with a control group was used. Participants were patients who applied for outpatient treatment and followed the intake procedure at the Human Concern Foundation, a specialized treatment center for eating disorders in the Netherlands. Data collection took place between March 2015 and January 2017. Inclusion criteria for this study were participants with (a) a minimum age of 16 years, which was the minimum age to apply for treatment at the treatment center, (b) a DSM 5 (American Psychiatric Association, 2013) ED diagnosis at intake, and (c) a signed informed consent. In total, 472 patients were diagnosed with an ED during the period of data collection and initially included in the study. However, after examining sex status, only four men were present in the study and excluded because of the very low sample size. In total 468 participants, all women were included in the final study. Patients were diagnosed by a psychiatrist in collaboration with an intake team consisting of a dietician, psychiatrist, or clinical psychologist, and a clinician with an eating disorder history who has been trained to use this experiential knowledge in treatment (de Vos et al., 2016). All patients filled in the questionnaires as part of the intake procedure and for the measurement baseline of Routine Outcome Monitoring (ROM). ROM is used during the treatment to monitor treatment progress. The following characteristics were collected during the intake interview and used in this study; presence of psychiatric history among family members in the first line (parents, brothers, sisters, medical classification), using psychotropic medication, having followed earlier treatments, being hospitalized earlier for the ED, duration, and start year of the ED, complex trauma, daily activities such as work or study and the financial situation (whether there were financial worries or actual financial problems). The intake interview consists of a semi-structured interview and is conducted by a licensed psychologist. Also, the answers which the patients have given are discussed during a meeting with the psychiatrist. Patients were asked whether they had experienced any life-events or trauma in the past. All answers which confirmed life-events or trauma were noted by the psychologist and/or psychiatrist. During the study the data was labeled by the researchers as life-events, trauma, or complex trauma (sexual abuse, verbal/physical abuse, severe instability in the family, or multiple negative life events). Only the label complex trauma was used in the study. The financial situation was explored with the following questions: are there any current financial problems, and, are there any current financial worries. All answers which confirmed financial problems or worries were noted and used by the researchers as an indication for a poor financial situation. Educational level and living situation were collected according to the instructions of Stichting Benchmark GGZ (SBG), a nationwide benchmark for treatment outcomes of mental health providers in the Netherlands (Stichting Benchmark GGZ., 2013). SBG was established in 2010 as an independent party to facilitate benchmarking among mental health providers in the Netherlands (Blijd-Hoogewys et al., 2012). All mental health institutions were obliged by mental health insurers to deliver data about their treatment outcomes and background variables of their patients, such as educational level and living situation. The results are used to compare outcomes (benchmarking) between treatment centers (Blijd-Hoogewys et al., 2012).

Patients were informed about the aims of the study and signed an informed consent stating that they could terminate the possibility to include their data for scientific research. Patients were consecutively selected based on application. This study required no additional data collection directly from patients apart from the regular intake procedure. Data was anonymized before data analysis. Only two patients declared that they did not want to have their data used in the study and were excluded. This study protocol was approved by the BMS Ethics Committee of the University of Twente.

To compare the well-being scores of ED patients with the general population, a control group of the LISS-panel (Longitudinal Internet Study in the Social Science) of CentERdata was used with a sample of 835 Dutch speaking non-institutionalized women from households in the Netherlands (Lamers et al., 2013). Data of the LISS-panel was collected between 2007 and 2008.

### Measures

#### Eating disorder psychopathology (EDE-Q)

Eating disorder psychopathology was measured with the original 36-item Eating Disorder Examination (EDE-Q), a widely used questionnaire for measuring ED psychopathology (Fairburn and Beglin, 1994). The scale consists of 22 items measuring the core attitudinal ED psychopathology. The global score is considered as a valid index of the general level of ED psychopathology (Aardoom et al., 2012). Patients rated the frequency of symptoms in the last 28 days using a 7-point Likert scale (0 = *not 1 day*; 6 = *every day*). The internal consistency of the global score in this sample was 0.92. Lower scores are indicative for lower ED psychopathology.

#### General psychopathology (OQ-45 symptomatic distress scale)

The scale symptomatic distress (SD scale) of the OQ-45 (Jong et al., 2008) was used as a measure for general psychopathology, in accordance with the Dutch benchmark for mental healthcare (Warmerdam et al., 2017). The OQ-45 SD scale has 25 items and shows good psychometric properties (Jong et al., 2008). Items are scored on a 5-point Likert scale, ranging from 0 “never” to 4 “always.” The internal consistency in this sample was 0.91. Higher scores are indicative of higher psychopathology.

#### Well-being (MHC-SF)

Well-being was measured with the Mental Health Continuum Short Form (MHC-SF). The Dutch MHC-SF was developed by Lamers et al. (2011) and includes emotional, psychological and social well-being. It consists of 14 items, rated on a six-point Likert scale ranging from 0 “never” to 5 “always,” and gives an overall impression of well-being. To examine whether the MHC-SF measures the same three dimensions of well-being in ED patients, as found in the general population (Lamers et al., 2011), confirmatory factor analysis (CFA) was tested prior to the main analysis in R statistics (R Core Team, 2013) with the Lavaan package version 0.5–23.1097 (Rosseel, 2012). The following fit indices were used: Root Mean Squared Error of Approximation (RMSEA), Comparative Fit Index (CFI), and Tucker-Lewis Index (TLI). CFA showed that a three-factor model, with the dimensions emotional, social, and psychological well-being, showed the best fit in our data, compared to a one or two factor model (RMSEA = 0.075, CFI = 0.93, and TLI = 0.92). The internal consistency in this sample was 0.90 for the total scale, 0.86 for the scale emotional well-being, 0.73 for the scale social well-being, and 0.83 for the scale psychological well-being. Higher scores are indicative of higher well-being.

The percentages of ED patients languishing, and flourishing were calculated according to Keyes (2002, 2012) and Lamers et al. (2011) instructions. For the category languishing, participants had to score low (“never” or “once or twice” during the past month) on at least one of the three emotional well-being dimensions and at least six of the eleven (combined) psychological and social well-being dimensions. For the category flourishing, participants had to score high (“almost every day,” or “every day” during the past month) on the same dimensions.

#### Severity indices of personality problems (SIPP-SF)

The SIPP-SF measures the core components of (mal)adaptive personality functioning in clinical samples using 60 items and is a shortened version of the SIPP-118 (Verheul et al., 2008). The SIPP measures the following components: self-control, identity integration, responsibility, relational capacities, and social concordance. Only the first three components were relevant for this study because the last two show a high overlap in construct and meaning with the social and psychological well-being dimensions. Self-control relates to the capacity to tolerate, use and control emotions and impulses. Identity integration relates to experiencing a coherence of self or identity and the capacity to see oneself and one's own life as stable, integrated, and purposive. Responsibility is related to taking responsibility for one's own life and the capacity to set realistic goals, and to achieve these goals (Verheul et al., 2008). The scales self-control, identity integration and responsibility all consist of 12 items and were rated from 1 “fully disagree” to 4 “fully agree.” Higher scores are indicative for lower levels of personality problems. The scales of the SIPP-SF show good construct validity and can be used to screen for (mal)adaptive personality functioning in adults (Verheul et al., 2008; Rossi et al., 2016). The internal consistency in this sample was 0.89 for the self-control/emotion regulation scale, 0.91 for the identity integration scale, and 0.86 for the responsibility scale. There was a lower response on the SIPP-SF (*N* = 269) because it was not administered in the first year of the data collection.

### Analysis

Analyses were performed in Statistical Package of the Social Sciences (SPSS) 24. Differences in mean levels of well-being between ED patients and the general population, and between AN, BN, BED, Other Specified Feeding and Eating Disorders (OSFED) and the general population were examined using one-way analysis of variance (ANOVA) with the groups as fixed factor. Partial eta-squared values (η^2^) were reported as measures for the effect size. Eta-squared values were interpreted as follows; low, ranging from 0.01 to 0.05, medium between 0.06 and 0.13, and large when 0.14 and higher (Field, 2005). Tukey's test was used for *post-hoc* analysis when homogeneity of variances was met. Welch's ANOVA and Games-Howell *post-hoc* tests were used when homogeneity of variances was not met.

Chi-square tests were used with *post-hoc* analysis using a Bonferroni correction to analyze differences in proportions flourishers and languishers between ED patients and the general population, and between the ED types. For correlation analysis Pearson coefficient was used. Correlations were interpreted as follows (Santrock, 2007): very low: 0 to 0.20 low: 21 to 0.40; moderate: 0.41–0.60; high moderate: 0.61–0.80; high: 0.81–0.90, and very high: 0.91–1.0. In addition, partial correlation analysis was run to determine whether possible confounders would lead to different results compared to Pearson correlation coefficient. The following control variables were used: age, living situation, educational level, duration of illness, start-age of the illness, financial situation, BMI, and whether patients had work or were following a study during the intake procedure. Although there were small changes when controlling for these variables (minimum difference 0.00, maximum difference 0.08), none led to changes in significance levels compared to Pearson correlation coefficient. For reasons of comparability with other research on the relationship between psychopathology and well-being, Pearson coefficient was used.

For the analysis of correlates that are expected to be associated with mental health, a multiple hierarchical regression analysis was used with the ED psychopathology and the well-being dimensions as dependent variables. Hierarchical regression was chosen because we used a broad set of potential correlates which could be classified into four main categories (1. demographics, 2. illness history related correlates, 3. current illness related correlates, and 4. (mal)adaptive personality functioning). Separate analyses were run on the following dependent variables; emotional well-being, psychological well-being, social well-being, and eating disorder psychopathology. Analysis of the three separate dimensions of well-being instead of only general well-being was done, because the dimensions are both theoretical and psychometrically different concepts of well-being (Deci and Ryan, 2008; Joshanloo and Lamers, 2016). The following predictor variables were used: demographic determinants: age, educational level, living situation, having a job or studying, and financial situation; illness history related determinants: ED duration, start age of ED, earlier hospitalization for the ED without remission, psychiatric history of a family member (1st degree), history with complex trauma; current illness-related determinants: eating disorder type, using psychotropic medication, general psychopathology, having frequent suicidal thoughts, Body Mass Index (BMI kg/m^2^) and personality related determinants: self-control/emotion-regulation, identity integration, and responsibility. Given the substantial number of independent variables in the regression analysis (*N* = 18), only *p*-values of < 0.01 were considered as significant determinants in the models.

Four hierarchical Models were used to test the stability of the associated correlates (independent variables) across the Models. In Model 1 the demographic variables were tested, in Model 2 the addition of illness-history-related variables to demographic variables were tested. In Model 3 and 4, respectively, the addition of current illness-related variables and (mal-)adaptive personality functioning were tested. The assessments of the Q-Q plots, partial regression plots, plots of studentized residuals, and the tolerance values below 0.1, indicated that the assumptions for linearity, homoscedasticity and normality were met, and that there was no multicollinearity.

## Results

### Background characteristics

In total 468 female patients with an average age of 28.4 years (*SD* = 9.9) participated in the study. Thirty-five patients (7.8%) had low education, 71 participants (15.8%) had intermediate education, and 343 (76.4%) had high education. The average start age of the ED was 16.0 years (*SD* = 5.0) and the average duration of the ED was 10.9 years (*SD* = 9.6). Hundred-sixty-one patients (34.4%) were diagnosed with Anorexia Nervosa (AN), 96 patients (20.5%) with Bulimia Nervosa (BN), 61 patients (13.0%) with Binge Eating Disorder (BED), and 150 patients (32.1%) with Other Specified Feeding or Eating Disorders (OSFED). The average start age and duration for AN was, respectively; 16.6 years (*SD* = 4.2) and 7.7 years (*SD* = 7.8), for BN; 15.5 years (*SD* = 4.9), and 11.6 years (*SD* = 8.6), for BED; 16.3 years (*SD* = 7.3) and 16.1 years (*SD* = 10.2) and for OSFED; 15.7 years (*SD* = 4.9) and 11.9 years (*SD* = 10.8). Eighty-five percent of the patients have received earlier psychiatric treatment. Mean scores on the EDE-Q global scale were 4.00 (*SD* = 1.19) for the overall ED sample, and 3.75 (*SD* = 1.25) for AN, 4.45 (*SD* = 0.92) for BN, 4.17 (*SD* = 1.01) for BED, and 3.93 (*SD* = 1.27) for OSFED. See the online Supplementary Material for an overview of the other background characteristics of the sample.

### Levels of well-being and percentages of flourishers and languishers

The first research question concerned the levels of well-being and the proportions of ED patients languishing and flourishing (categorical scores) compared to the general population. There were statistically significant differences between ED patients and the general population for overall well-being, *Welch's F*_(1, 884.14)_ = 188.55, *p* < 0.001, emotional well-being, *Welch's F*_(1, 800.779)_ = 464.89, *p* < 0.001, psychological well-being, *F*_(1, 1301)_ = 199.78, *p* < 0.001, and social well-being *F*_(1, 1301)_ = 13.83, *p* < 0.001. ED patients had lower average scores compared to the general population on all well-being scales with medium effect sizes for overall (η^2^ = 0.13) and psychological well-being (η^2^ = 0.13), a low effect size for social well-being (η^2^ = 0.01) and a large effect size for emotional well-being (η^2^ = 0.29). See Table 1 for an overview of the results. *Post-hoc* analysis showed statistically significant lower well-being scores for all ED types compared to the general population on overall (*p* < 0.001), emotional (*p* < 0.001), and psychological well-being (*p* < 0.001). Social well-being was only statistically significant lower for AN (*p* < 0.05), and BN (*p* < 0.05) compared to the general population. Differences between the ED types were only found between AN and BED for emotional (*p* < 0.01) and psychological well-being (*p* < 0.05).

**Table 1:** Categorical and mean scores of well-being for the general population and ED patients.

	**Groups**							**ED Types**									
	**General population (*****N*** = **835, women)**		**ED patients (*****N*** = **468)**		**Statistics**			**AN (*****N*** = **161)**		**BN (*****N*** = **96)**		**BED (N** = **61)**		**OSFED (*****N*** = **150)**		**Statistics**	
	***N***	**%**	***N***	**%**	**χ^2^**	***_*p*_***		***N***	**%**	***N***	**%**	***N***	**%**	***N***	**%**	**χ^2^**	***_*p*_***
**MENTAL HEALTH CATEGORIES**																
					169.327	.000										17.204	.009
Languishing	40	4.8%	122	26.1%**				54	33.5%*	27	28.1%	12	19.7%	29	19.3%
Moderate	488	58.4%	285	60.9%				92	57.1%	59	61.5%	34	55.7%	100	66.7%
Flourishing	307	36.8%	61	13.0%**				15	9.3%	10	10.4%	15	24.6%*	21	14%
	***M***	***SD***	***M***	***SD***	***F***	***p***	*η^2^*	***M***	***SD***	***M***	***SD***	***M***	***SD***	***M***	***SD***	***F***	***p***
**WELL-BEING**																
Total	3.00	0.84	2.29	0.94	188.555	0.000	.13	2.17	0.94	2.21	0.92	2.52	1.02	2.36	0.89	52.632	0.000
Emotional	3.70	0.92	2.36	1.16	464.899	0.000	.29	2.16	1.25	2.34	1.04	2.79	1.12	2.40	1.10	119.922	0.000
Psychological	3.22	0.98	2.40	1.04	199.780	0.000	.13	2.25	1.04	2.32	1.03	2.68	1.12	2.50	0.99	52.803	0.000
Social	2.32	1.02	2.10	1.00	13.836	0.000	.01	2.07	0.93	2.00	1.06	2.18	1.12	2.17	0.99	4.018	0.003

* Statistically significant different at post-hoc comparisons at the p < 0.01 level with Bonferroni correction,

** *Statistically significant different at post-hoc comparisons at the p < 0.001 level, with Bonferroni correction, percentages are rounded to the nearest tenth*.

Sixty-one patients in this sample were flourishing (13.0%), 285 patients had moderate well-being (60.9%), and 122 patients (26.1%) were languishing. Compared to the general population, there were statistically significant differences in the proportions of ED patients who were languishing, had moderate well-being and were flourishing, $\chi_{\left(2\right)}^{2}$ = 169.327, *p* < 0.001. *Post hoc* comparisons of categorical scores showed that statistically significantly more patients with AN were languishing (33.5%, *p* < 0.001) and more patients with BED were flourishing (24.6%, *p* < 0.001), compared to the other ED types.

These results confirm our hypothesis that ED patients show lower levels of well-being and have a higher change to languish compared to the general population. Substantial variation was found in ED patients' well-being, while all were diagnosed with severe psychopathology. These results suggest that psychopathology and well-being are two related but distinct continua of mental health.

### Relationship of psychopathology with well-being

The second research question concerned the correlation between psychopathology and well-being. Table 2 shows the correlations (Pearson's r) between psychopathology and well-being for ED patients overall and the specific ED types.

**Table 2:** Pearson correlation between psychopathology and well-being per eating disorder type.

**ED type**	**Psychopathology**			**Well-being**		
		**General path**	**Total**	**Emotional**	**Psychological**	**Social**
Total	ED path	0.43*	−0.35*	−0.33*	−0.33*	−0.29*
	General path	–	−0.73*	−0.71*	−0.69*	−0.55*
AN	ED path	0.52*	−0.53*	−0.48*	−0.54*	−0.41*
	General path	–	−0.75*	−0.74*	−0.72*	−0.58*
BN	ED path	0.48*	−0.20	−0.20	−0.15	−0.18
	General path	–	−0.69*	−0.62*	−0.64*	−0.57*
BED	ED path	0.22	−0.07	−0.08	−0.00	−0.14
	General path	–	−0.72*	−0.72*	−0.69*	−0.56*
OSFED	ED path	0.42*	−0.39*	−0.38*	−0.37*	−0.29*
	General path		−0.72*	−0.72*	−0.68*	−0.51*

* *correlation is significant at the 0.001 level (2 sided), ED path = Eating Disorder psychopathology; General path = General psychopathology*.

General psychopathology showed a *high moderate negative* correlation with overall (*r* = −0.73, *p* < 0.001), and emotional well-being (*r* = −0.71, *p* < 0.001), and a *moderate negative* correlation for psychological (*r* = −0.69, *p* < 0.001) and social well-being (*r* = −0.55, *p* < 0.001).

ED psychopathology showed a *low negative* correlation with overall (*r* = −0.35, *p* < 0.001), emotional (*r* = −0.33, *p* < 0.001), psychological (*r* = −0.33, *p* < 0.001), and social well-being (*r* = −0.29, *p* < 0.001).

Analysis per ED type showed *moderate* to *high moderate negative* correlations of general psychopathology with all well-being dimensions for AN, BN, BED, and OSFED (*p* < 0.001). For ED psychopathology however, only significant *low to moderate negative* correlations with well-being dimensions were found for AN and OSFED (*p* < 0.001), while for BN and BED no significant correlation was found with any well-being dimension.

The results suggest that ED psychopathology and well-being are two related but distinct continua, whereas general psychopathology and well-being are two separate but stronger related continua. Overall, our hypothesis was confirmed.

### Correlates with mental health

The third question concerned correlates for psychopathology and well-being. See Table 3 for each regression model, and Table 4 for the tested variables in the hierarchical multiple regression.

**Table 3:** Hierarchical Multiple Regression with four models consisting of variables with: 1. Demographics, 2. Illness history, 3. Current illness, and 4. Personality traits.

**Model**	***R*^2^**	***F***	**Sig**	***R*^2^ change**	***F* change**	**Sig change**
**ED PSYCHOPATHOLOGY**						
Model 1	0.032	1.216	0.295	–	–	–
Model 2	0.045	0.976	0.472	0.013	0.653	0.659
Model 3	0.271	5.276	0.000	0.226	10.700	0.000
Model 4	0.281	4.724	0.000	0.010	1.103	0.348
**EMOTIONAL WB**						
Model 1	0.053	2.023	0.053	–	–	–
Model 2	0.066	1.476	0.133	0.014	0.726	0.604
Model 3	0.522	13.887	0.000	0.455	32.894	0.000
Model 4	0.596	16.051	0.000	0.075	14.757	0.000
**PSYCHOLOGICAL WB**						
Model 1	0.056	2.169	0.037	–	–	–
Model 2	0.076	1.703	0.067	0.019	1.047	0.390
Model 3	0.497	12.609	0.000	0.422	29.009	0.000
Model 4	0.642	19.460	0.000	0.144	32.080	0.000
**SOCIAL WB**						
Model 1	0.061	2.374	0.023	–	–	–
Model 2	0.069	1.544	0.109	0.008	0.419	0.835
Model 3	0.342	6.580	0.000	0.272	14.299	0.000
Model 4	0.444	8.685	0.000	0.103	14.720	0.000

*Model 1: Demographics: age, educational level, living situation, having a job or study, and financial situation, Model 2: model 1 + Illness history: ED duration, start age of ED, earlier inpatient treatment(s) without remission, psychiatric history of a family member (1st degree), having complex trauma, Model 3: model 2 + Current Illness variables: eating disorder type, using psychotropic medication, general psychopathology, having frequent suicidal thoughts, Body Mass Index (BMI kg/m^2^), Model 4: model 3 + Personality traits: self-control/emotion-regulation, identity integration, responsibility*.

**Table 4:** Hierarchical multiple regression: correlates associated with ED psychopathology and well-being.

**Variable**	**Psychopathology**			**Well-being**								
	**Eating disorder**			**Emotional**			**Social**			**Psychological**		
	**B (CI 95%)**	***SE***	**β**	**B (CI 95%)**	***SE***	**β**	**B (CI 95%)**	***SE***	**β**	**B (CI 95%)**	***SE***	**β**
**BLOCK 1 DEMOGRAPHICS**												
Age	−0.018 (−0.047–0.011)	0.015	−0.151	0.002 (−0.019–0.023)	0.011	0.021	−0.002 (−0.023–0.020)	0.011	−0.015	−0.014 (−0.032–0.004)	0.009	−0.135
Low education	0.312 (−0.504–1.127)	0.414	0.069	−0.084 (−0.678–0.511)	0.302	−0.019	−0.135 (−0.737–0.466)	0.306	−0.036	−0.342 (−0.845–0.160)	0.255	−0.087
Intermediate education	0.173 (−0.565–0.911)	0.375	0.052	−0.225 (−0.763–0.312)	0.273	−0.070	−0.428 (−0.972–0.118)	0.277	−0.153	−0.477 (−0.932–−0.022)	0.231	−0.165
High education	0.068 (−0.607–0.743)	0.343	0.025	−0.231 (−723–0.260)	0.250	−0.089	−0.248 (−0.746–0.250)	0.253	−0.110	−0.325 (−0.741–0.091)	0.211	−0.138
Living situation	−0.104 (−0.385–0.177)	0.143	−0.042	0.018 (−0.187–0.225)	0.104	0.007	0.097(−0.111–0.304)	0.105	0.047	0.068 (−0.105–0.241)	0.088	0.032
Main activity	−0.110 (−0.441–0.221)	0.168	−0.039	0.017 (−0.224–0.258)	0.122	0.006^a^, ^b^	−0.082 (−0.326–0.162)	0.124	−0.035^a^, ^b^	0.078 (−0.126–282)	0.104	0.032^a^, ^b^
Financial situation	0.083 (−0.382–0.548)	0.236	0.020	−0.008 (−0.347–0.331)	0.172	−0.002	−0.061 (−0.404–0.282)	0.174	−0.018	0.004 (−0.283–0.291)	0.146	0.001
**BLOCK 2 ILLNESS HISTORY**												
ED duration	0.002 (−0.028–0.032)	0.015	0.015	−0.004 (−0.025–0.018)	0.011	−0.029	−0.002 (−0.024–0.020)	0.011	−0.017	0.005 (−0.013–0.024)	0.009	0.050
Start age ED	0.007 (−0.029–0.042)	0.018	0.028	−0.004 (−0.030–0.021)	0.013	−0.018	−0.006 (−0.032–0.020)	0.013	−0.031	−0.004 (−0.025–0.018)	0.011	−0.017
Earlier hospitalized	0.145 (−0.197–0.488)	0.174	0.050	−0.189 (−0.438–0.061)	0.127	−0.067	−0.245 (−0.497–0.008)	0.128	−0.101	−**0.380 (**−**0.591–**−**0.169)**	**0.107**	0.150***
Psychiatric family	−0.067 (−0.348–0.214)	0.143	−0.028	0.006 (−0.211–0.199)	0.104	0.003	0.031 (−0.177–0.339)^c^	0.105	0.015	−0.067 (−0.240–0.107)	0.088	−0.032
Complex trauma	0.198 (−0.247–0.644)	0.226	0.051	−0.216 (−0.514–0.109)	0.165	−0.057	−0.062 (−0.391–0.267)	0.167	−0.019	−0.004 (−0.279–0.271)	0.139	−0.001
**BLOCK 3 CURRENT ILLNESS**												
AN	−**0.832 (**−**1.378–**−**0.286)**	**0.277**	−0.332**	−0.114 (−0.511–0.265)	0.201	−0.047	0.037 (−0.366–0.440)	0.205	0.018	0.033 (−0.304–0.369)	0.171	0.015
BN	−0.012 (−0.502–0.478)	0.249	−0.004	−0.192 (−0.549–0.165)	0.181	−0.067	−0.134 (−0.495–0.228)	0.184	−0.054	−0.126 (−0.428–0.177)	0.153	−049
BED	−0.448 (−0.913–0.016)	0.236	−0.176	−0.243 (−0.581–0.096)	0.172	−0.098	−0.068 (−0.411–0.274)	0.174	−0.032	−0.119 (−0.405–168)	0.145	−0.053
BMI	0.004 (−0.019–0.027)	0.0011	0.026	−0.004 (−0.020–0.013)	0.008	−0.025	−0.015 (−0.032–002)	0.008	−0.114^c^	0.003 (−0.011–0.017)	0.007	0.020
Psychotropic medication	0.156 (−0.179–0.492)	0.170	0.054	−0.071 (−0.315–0.174)	0.124	−0.025	0.069 (−0.178–0.317)	0.126	0.019	0.117 (−0.089–0.324)	0.105	0.046
General psychopathology	**0.035** (**0.016**–**0.054)**	**0.010**	0.413***	−**0.020 (**−**0.034–**−**0.006)**	**0.007**	−0.236*	0.000 (−0.015–0.014)	0.007	−0.007^c^	0.002 (−0.012–0.016)	0.007	−0.015^c^
Frequent suicidal thoughts	−0.010 (−0.495–0.476)	0.246	−0.002	−0.079 (−0.432–0.275)	0.180	−0.020	0.217 (−0.141–0.575)	0.182	0.063	0.262 (−0.037–0.562)	0.152	0.074
**BLOCK 4 PERSONALITY TRAITS**												
Self–control/emotion regulation	0.004 (−0.022–0.029)	0.013	0.021	−0.006 (−0.024–0.013)	0.009	−0.034	0.002 (−0.017–021)	0.013	0.017	−0.006 (−0.022–0.010)	0.008	−0.041
Identity integration	−0.010 (−0.042–0.022)	0.016	−0.071	**0.079** (**0.056**–**0.103)**	**0.012**	0.575***	**0.071 (0.047** −**0.095)**	**0.012**	0.600***	**0.088 (0.068–0.108)**	**0.010**	0.711***
Responsibility	0.019 (−0.007–0.046)	0.013	0.105	−0.008 (−0.027–0.012)	0.010	−0.043	0.021 (0.002–0.041)	0.010	0.137	**0.031 (0.015–0.048)**	**0.008**	0.195***

Significant correlates in the final model are highlighted in bold,

* p = < 0.001 *p = < 0.01,

** p = < 0.001,

*** P < 0.0001.

a Significant predictor in model 1;

b Significant predictor in model 2;

c Significant predictor in model 3.

*Model 1 Demographics: age, educational level, living situation, having a job or study and financial situation, Model 2: model 1 + Illness history: ED duration, start age of ED, earlier inpatient treatment(s) without remission, psychiatric history of a family member (1st degree), having complex trauma. Model 3: model 2 + Current Illness variables: eating disorder type, using psychotropic medication, general psychopathology, having frequent suicidal thoughts, Body Mass Index (BMI kg/m^2^). Model 4: model 3 + Personality traits: self-control/emotion-regulation, identity integration, responsibility*.

#### Correlates associated with ED psychopathology

The explained variance of the baseline Model (1) with demographic variables related to ED psychopathology was not statistically significant [*R*^2^ = 0.032, *F*_(7, 254)_ = 1.21, *p* > 0.01]. The addition of illness-history-related variables correlated to ED psychopathology (Model 2) above the standard demographic variables (Model 1) did not lead to a statistically significant increase in *R*^2^ = 0.013, *F*_(7, 254)_ = 0.653, *p* > 0.01. Explained variance increased significantly with the addition of current illness-related variables relating to ED psychopathology (Model 3): *R*^2^ = 0.226, *F*_(19, 242)_ = 10.70, *p* < 0.001. The addition of personality-related variables relating to ED psychopathology (Model 4) did not lead to a statistically significant increase in *R*^2^ = 0.010, *F*_(22, 239)_ = 1.10, *p* > 0.01. Model 3 showed the best fit determining ED psychopathology [*R*^2^ = 0.281, *F*_(19, 242)_ = 4.238, *p* < 0.001]. AN (β = −0.332, *p* < 0.01) and general psychopathology (β = 0.413, *p* < 0.001) was significantly associated with ED psychopathology.

#### Correlates associated with emotional well-being (EWB)

The explained variance of the baseline Model (1) with demographic variables relating to EWB was not statistically significant [*R*^2^ = 0.053, *F*_(7, 254)_ = 2.023, *p* > 0.01]. The addition of illness-history-related variables relating to EWB (Model 2) above the standard demographic variables (Model 1) did not lead to a statistically significant increase in *R*^2^ = −0.014, *F*_(7, 254)_ = 0.726, *p* > 0.01. Explained variance increased significantly with the addition of current illness related variables relating to EWB (Model 3): *R*^2^ = 0.455, *F*_(19, 242)_ = 32.894, *p* < 0.001. The addition of personality related variables relating to EWB (Model 4) showed a statistically significant increase in *R*^2^ = 0.075, *F*_(22, 239)_ = 14.757, *p* < 0.001. The final model (4) showed the best fit to determine EWB [*R*^2^ = 0.596, *F*_(22, 239)_ = 16.051, *p* < 0.001]. General psychopathology (β = −0.236, *P* < 0.01), and identity integration (β = 0.575, *p* < 0.001) were significantly associated with EWB.

#### Correlates associated with psychological well-being (PWB)

The explained variance of the baseline Model (1) with demographic variables relating to PWB was statistically significant [*R*^2^ = 0.056, *F*_(7, 254)_ = 2.16, *p* < 0.05]. The addition of illness-history-related variables relating to PWB (Model 2) above the standard demographic variables (Model 1), did not lead to a significant increase in *R*^2^ of 0.019, *F*_(7, 254)_ = 1.05, *p* > 0.05. The addition of current illness-related variables relating to PWB (Model 3) led to a statistically significant increase in *R*^2^ of 0.422, *F*_(19, 242)_ = 29.01, *p* < 0.001. The addition of personality-related variables relating to PWB (Model 4) led to a statistically significant increase in *R*^2^ of 0.144, *F*_(22, 239)_ = 33.08, *p* < 0.001. The final Model (4) showed the best fit to determine PWB [*R*^2^ = 0.642, *F*_(22, 239)_ = 19.46, *p* < 0.001]. Specific correlates for PWB were, an Earlier hospitalization (β = 0.150, *p* < 0.001), and the personality traits identity integration (β = 0.711, *p* < 0.001), and responsibility (β = 0.195, *p* < 0.001).

#### Correlates associated with social well-being (SWB)

The baseline Model (1) with demographic variables relating to SWB was not statistically significant [*R*^2^ = 0.061, *F*_(7, 254)_ = 2.37 *p* > 0.05]. The addition of illness history related variables relating to SWB (model 2) above the standard demographic variables (Model 1), led not to a statistically significant increase in *R*^2^ of 0.008, *F*_(7, 254)_ = 0.419, *p* > 0.05. The addition of current illness-related variables relating to SWB (Model 3) showed a statistically significant increase in *R*^2^ of 0.272, *F*_(19, 242)_ = 14.23, *p* < 0.001. The addition of personality-related variables relating to SWB (Model 4) led to a statistically significant increase in *R*^2^ of 0.103, *F*_(22, 239)_ = 14.72, *p* < 0.001. The final Model (4) showed the best fit to determine SWB [*R*^2^ = 0.444, *F*_(22, 239)_ = 8.69, *p* < 0.001]. The personality trait identity integration (β = 0.600, *p* < 0.001) was significantly associated with SWB.

Our hypothesis can be partly confirmed, as there were different correlates associated with ED psychopathology (having AN) and well-being (earlier hospitalization for the ED, identity integration, and responsibility). However, general psychopathology was associated with both ED psychopathology and emotional well-being.

## Discussion

This is one of the first studies to examine all dimensions of well-being (emotional, psychological, and social) in female eating disorder patients. The levels of well-being as well as the derived percentages of languishing and flourishing were compared to the general population. Also, the relationship of well-being with psychopathology and correlates for both were examined. Overall, results indicate a two-continuum structure of mental health where psychopathology and well-being are negatively related. As expected, ED patients in this sample had lower levels of well-being compared to the general population. There was also substantial variation in the levels of well-being among ED patients, despite the high levels of psychopathology, indicating that well-being should be considered as a distinct mental health continuum. The two continua model is further supported by the fact that there were no high correlations between ED psychopathology and well-being and that different correlates were associated with ED psychopathology and well-being.

### The presence of well-being in ED patients

In this study we found overall lower mean levels of emotional, psychological, and social well-being among female ED patients compared to the general female population. In particular, a large difference was found for emotional well-being, with ED patients scoring the lowest levels compared to controls. The percentage of ED patients languishing (26.1%) was substantially higher and the percentage of flourishing substantially lower (13.0%) compared to the general population (respectively, 4.8 and 36.8%). These results correspond with earlier work of Tomba et al. (2014), who found lower levels of psychological well-being among ED patients at the start of outpatient treatment compared to general controls. The two continua model of mental health suggests that clinical populations on average will have lower levels of well-being and higher levels of psychopathology compared to the general population, but that a part may still have moderate or high levels of well-being. Although the patients in this study have been diagnosed with a severe psychiatric disorder, are seeking specialized ED treatment and show on average a long duration (10.9 years) of the ED, a substantial part of them still functions moderately well on well-being (60.9%), and a small part (13%) even shows high well-being (flourishing). It is important to further examine why some patients are still able to experience adequate levels of well-being despite having an ED.

Our research showed interesting differences between ED types. We found that languishing was most prevalent in patients with AN (33.5%) and that flourishing was most prevalent in patients with BED (about 24.6%). This seems contrary to the results of Tomba et al. (2014), who found that patients with AN were the most similar to “healthy” controls on the dimensions of psychological well-being, while patients with BN showed the most impairment compared to controls. They related the relative good outcomes of AN patients to possible ego syntonic aspects of the disease, a lack of insight, their ability of controlling weight, socio-cultural values, such as thinness desirability and personality traits (Tomba et al., 2014). This study suggests that patients with AN are vulnerable to lower overall well-being. It is possible that our sample of patients with AN was more severe in terms of lower BMI (16.1 kg/m^2^ compared to 17.5 kg/m^2^ in Tomba et al., 2014), a longer illness duration, or higher ED psychopathology. A recent review on the ego syntonic nature of anorexia nervosa showed that in the initial phases of the disease patients may experience a sense of mastery and self-control for being able to manage food and to reach weight-related goals, and in addition they may attain confidence and self-worth as a result of positive feedback from family and friends (Gregertsen et al., 2017). These advantages are primarily related to the dimensions of psychological well-being and reinforce the ego syntonic nature of the disease. However, as the disease progresses and begins to take over their thoughts and behavior, these patients begin to lose control, rather than be in control (Gregertsen et al., 2017). The consequences of the disease, such as physical health issues, negative changes in brain functioning, negative instead of positive feedback from family and friends, social isolation, and psychological problems, become more apparent and are beginning to outweigh the advantages (Gregertsen et al., 2017). It is plausible that patients with AN who are longer in the process of the disease and are beginning to outweigh these disadvantages above the advantages will perceive lower levels of well-being, while patients in the initial phases of the disease on the other hand will experience higher levels of well-being. Therefore, the levels of well-being in patients with AN might be determined by the duration of the ED, ego syntonic aspects, and physical consequences, which should be a topic for further study.

Patients with BED, on the other hand, showed higher well-being compared to the other ED types, but also a long average duration of the ED (16.1 years). It has been suggested that binge eating should be considered as a coping mechanism for perceived daily stress (Freeman and Gil, 2004). A possible explanation for BED patients is that using binges, without extreme compensating behaviors, could be considered as a relatively effective coping mechanism for internal and external stressors. Therefore, BED patients would be able to maintain positive functioning in their life and maintain higher levels of well-being, despite a long-term ED. Another reason might be that in general, the brain of patients with BED receives enough nutrition to be able to experience forms of well-being. For patients with AN the severe implications of prolonged malnutrition alters brain functioning and mental health (Fuglset et al., 2016), which might also contribute to the presence of lower well-being later in the illness process.

### The relationship of well-being with psychopathology

General psychopathology had negative moderate to high moderate correlations, and ED psychopathology had low correlations with the well-being dimensions. Moderate correlations between general psychopathology and well-being were also found in other clinical populations (Schotanus-Dijkstra et al., 2015; Franken et al., 2018). Contrary to our expectations, the correlations between ED psychopathology and well-being were substantially lower compared to the correlations between general psychopathology and well-being. It seems that ED psychopathology functions relatively independent from the well-being levels. This may have important implications for treatment and suggests that a focus only on alleviating ED psychopathology in treatment does not necessarily adequately improve well-being among ED patients and contrariwise. Alleviating general psychopathology however, might both improve emotional well-being and alleviate ED psychopathology. While there were no differences between the correlations of general psychopathology and well-being between ED types, for ED psychopathology there were low to moderate correlations for patients with AN, OSFED and overall ED's, and no correlations with the well-being dimensions for patients with BN and BED. This was also unexpected, and we have no clear explanation for these results. It is suggested to perform qualitative research to further explore these relationships among ED patients and examine this complex relationship between psychopathology and well-being and possible reciprocal relationships in a longitudinal study design to make more reliable conclusions on correlates between ED psychopathology and well-being.

### Correlates associated with ED psychopathology and well-being

Our research showed that different correlates were associated with well-being and psychopathology. Having AN and lower general psychopathology were associated with lower ED psychopathology. This probably has to do with how ED psychopathology is measured, namely with the EDE-Q. It is found that outpatients with AN score significantly lower on the global score compared to patients with BN (Dahlgren et al., 2017). In this study patients with AN scored also substantially lower on the EDE-Q global score compared to the other ED types in this study. The association of higher general psychopathology with higher ED psychopathology is well supported by other research (Jacobi et al., 2004; Spindler and Milos, 2007).

There were several associated correlates for emotional, psychological, and social well-being. Patients with higher general psychopathology and a lower identity integration had a higher change on poorer emotional well-being. Patients with a history of hospitalization for the ED and a lower score on the personality traits, responsibility, and identity integration had a higher change of poorer psychological well-being. Finally, lower identity integration was also associated with lower levels of social well-being. The importance of personality traits, more specific conscientiousness and extraversion as predictors for adequate well-being was also found in the general population (Schotanus-Dijkstra et al., 2015). Responsibility is related to conscientiousness and is related to setting goals and maintaining the discipline necessary to achieve those goals. This may suggest that patients who have learnt to take responsibility for their own recovery process, for instance, by doing the homework provided in treatment, will have a higher change to improve on well-being dimensions during treatment. Previous research shows that disturbances in overall identity development are a core vulnerability for ED pathology (Farchaus Stein and Corte, 2007), and it is suggested that the development of a positive self may be an important factor in ED recovery. Further research is needed to examine the complex relationship between identity impairment, well-being, and ED pathology. Also, these correlates should be examined in a longitudinal design to draw conclusions on whether they might predict changes in ED psychopathology and well-being later in treatment.

It is notable that being hospitalized earlier in life for an ED was associated with lower psychological well-being, while it was not associated with more severe ED psychopathology. Patients who have been admitted once for ED symptoms, often need at least one readmission, which enhances the risk of later psychosocial functioning (Steinhausen and Grigoroiu-serbanescu, 2008). In a longitudinal study, readmission showed no effect on ED symptoms at 8 years follow-up, however, there was clear evidence that patients with readmissions functioned less well in terms of psychosocial adaptation (Steinhausen and Grigoroiu-serbanescu, 2008). Hospitalization for an ED is possibly a specific negative life event, even when it was not compulsory, impacting a patient's psychological well-being. It is therefore warranted, to further study the best way to deliver these treatments, and to carefully consider and monitor the possible impact afterwards on the psychological well-being of patients.

### Strengths and limitations

The strengths of this study are the large sample of ED outpatients and the large control group. The limitations of this study are its design, having only an adult female sample, the lack of registration of specific co-morbidity and other possible confounders, the use of self-report instruments, the sample size of patients with BED, and the arbitrary criteria for categorizing levels of well-being. A cross-sectional design was used, meaning that no causal inferences can be made and that longitudinal study designs are necessary for more conclusive results. No adolescents younger than 16 and no males were included in this sample and results are therefore not generalizable to these populations. Self-report instruments were used, making it susceptible to biases, such as social desirability bias (Atkinson et al., 1997; van de Mortel, 2008). However, to date no other valid, generally recognized methods for examining well-being were available. The relatively low sample size of patients with BED, compared to patients with AN, BN, and OSFED is a limitation. Also, while in this study the severity of general psychopathology was reported, specific co-morbidity was not reported, as well as possible other confounders, such as physical activity and body image distortion.

The criteria for flourishing and languishing used in this study should be considered arbitrary, and there are other approaches, based on other questionnaires and categorical scorings to examine flourishing, which are not necessarily comparable (Schotanus-Dijkstra et al., 2015). Keyes (2005) operationalized the clusters of components for well-being (i.e., emotional, psychological, social) in the same way as the clusters of symptoms for mental disorders in the Diagnostic and Statistical Manual of Mental Disorders (DSM) (American Psychiatric Association, 2000). Just as the diagnosis of depression requires symptoms of anhedonia, well-being requires “symptoms” of emotional vitality and positive feelings toward one's life (Keyes, 2005). Keyes (2005) also proposed to use a categorical scoring, based on the DSM approach. Where depression is diagnosed when at least one symptom from the anhedonia cluster and four or more symptoms of malfunctioning are present, well-being is diagnosed when at least one “symptom” of emotional well-being and six “symptoms” of psychological and social well-being are present. However, the criteria and categorical scoring of the DSM mental disorders are based on consensus by expert workgroups, there is no such procedure orconsensus yet for establishing (cut-off) criteria for adequate well-being. It is advised to form expert workgroups to seekconsensus on the categories and cut-off criteria for determining languishing and flourishing. At last, in this study no separate analysis was done on patients between 16 and 18, compared to adolescents.

## Conclusions and clinical implications

Overall the results of this study have some important implications. This study shows initial support for the two continua model for mental health among ED patients. ED patients have lower levels of well-being compared to the general population, however a small part is also flourishing. Examining the levels of well-being at the start of treatment is therefore advised. A broader vision on mental health, such as the two continua model among clinical samples, may open up pathways to reduce mental health stigma. The model could support a broader focus in psychological treatments on relevant dimensions of mental health such as well-being, instead of an almost exclusive focus on the diagnostic label and underlying psychopathology. For instance, study results show that health professionals were often described by patients and their family as the source of stigma, frequently focusing on the illness and ignoring other aspects of the patient (Corrigan et al., 2014).

A focus in treatment on well-being should be considered for those patients with inadequate well-being. A focus *only* on alleviating ED psychopathology in treatment might inadequately improve well-being, given the overall low to moderate strengths of the correlations. This might be even more important to consider in specific ED subtypes such as BN and BED, where no significant correlations between both were found. Several correlates were found to be associated with ED psychopathology and well-being. A focus on alleviating general psychopathology is advised and might decrease ED psychopathology and increase emotional well-being. Treatments should aim at increasing personality functioning on identity integration and responsibility and prevent hospitalization for ED if possible, because this will have a positive effect on well-being among ED patients.

## Ethics statement

This study was carried out in accordance with the recommendations of the code of ethics for research in the social and behavioral sciences involving human participants in the Netherlands (WMO, 2016). The protocol was approved by the Ethics Committee of the University of Twente, Behavioral, Management and Social Sciences. All subjects gave written informed consent in accordance with the Declaration of Helsinki.

## Author contributions

JdV wrote on all parts of the manuscript (introduction, methods, results, discussion), did the data collection and analysis. MR wrote on all parts of the manuscript and tested and supervised the analysis. EB and GW wrote on most parts of the manuscript, validated the analysis, and supervised the complete study.

### Conflict of interest statement

The authors declare that the research was conducted in the absence of any commercial or financial relationships that could be construed as a potential conflict of interest.
