# Supplementary material for: Having an Eating Disorder and Still Being Able to Flourish? Examination of Pathological Symptoms and Well-Being as Two Continua of Mental Health in a Clinical Sample
Source: Front Psychol. 2018 Nov 15;9:2145. doi: 10.3389/fpsyg.2018.02145 (PMC6249270; doi:10.3389/fpsyg.2018.02145)
Supplement: Supplementary file 1 [file Table_1.docx]

Supplementary table: Patient characteristics per eating disorder type

| Measures/Characteristics |  | | ED Types | |  | | |  | | | |  |  |  |
| --- | --- | --- | --- | --- | --- | --- | --- | --- | --- | --- | --- | --- | --- | --- |
|  | ED total  (N = 468) | | AN  (*N* = 161) | | | BN  (*N* = 96) | | | BED (N = 61) | | OSFED  (*N* = 150) | | | |
|  | *M* | *(SD)* | *M* | (*SD*) | | *M* | (*SD*) | | *M* | (*SD*) | | *M* | (*SD*) |  |
| Age | 28.4 | (9.9) | 25.6 | (8.2) | | 29.0 | (9.5) | | 33.2 | (11.1) | | 29.0 | (10.3) |  |
| BMI (kg/m^2^) | 21.6 | (7.7) | 16.1 | (1.8) | | 23.2 | (6.3) | | 30.3 | (8.3) | | 23.1 | (7.8) |  |
| Start of ED (Age) | 16.0 | (5.0) | 16.6 | (4.2) | | 15.5 | (4.9) | | 16.3 | (7.3) | | 15.7 | (4.9) |  |
| ED Duration | 10.9 | (9.6) | 7.7 | (7.8) | | 11.6 | (8.6) | | 16.1 | (10.2) | | 11.9 | (10.8) |  |
|  | *N* | *(%)* | *N* | *(%)* | | *N* | *(%)* | | *N* | *(%)* | | *N* | *(%)* |  |
| Education | *N* = 19 missing | | *N* = 5 missing | | | *N* = 4 missing | | | *N* = 3 missing | | | *N* = 7 missing | |  |
| Low | 35 | (7.8%) | 15 | (9.6%) | | 9 | (9.8%) | | 2 | (3.5%) | | 9 | (6.3%) |  |
| Intermediate | 71 | (15.8%) | 31 | (19.9%) | | 12 | (13%) | | 10 | (16.4%) | | 18 | (12.6%) |  |
| High | 343 | (76.4%) | 110 | (70.5%) | | 71 | (77.2%) | | 46 | (79.3%) | | 116 | (81.1%) |  |
| Living situation | *N* = 16 missing | | *N* = 5 missing | | | *N* = 4 missing | | | *N* = 2 missing | | | *N* = 5 missing | |  |
| Single | 165 | (36.5%) | 49 | (31.4%) | | 40 | (43.5%) | | 31 | (52.5%) | | 45 | (31%) |  |
| Single parent (with children) | 12 | (2.7%) | - | - | | 7 | (7.6%) | | 3 | (2.1%) | | 2 | (3.4%) |  |
| With partner, without children | 70 | (15.5%) | 17 | (10.9%) | | 15 | (16.3) | | 9 | (15.3%) | | 29 | (20%) |  |
| With partner, with children | 47 | (10.4%) | 11 | (7.1%) | | 6 | (6.5%) | | 10 | (16.9%) | | 20 | (13.8%) |  |
| Daughter in a one parent family | 19 | (4.2%) | 10 | (6.4%) | | 4 | (4.3%) | | - | - | | 5 | (3.4%) |  |
| Daughter in a double parent family | 112 | (24.8%) | 60 | (38.5%) | | 14 | (15.2%) | | 5 | (8.5%) | | 33 | (22.8%) |  |
| Other | 27 | (6.0%) | 9 | (5.8%) | | 6 | (6.5%) | | 2 | (3.4%) | | 10 | (6.9%) |  |
|  |  |  |  |  | |  |  | |  |  | |  |  |  |
| Earlier psychiatric treatment | 398 | (85.0%) | 137 | (85.1%) | | 78 | (81.3%) | | 55 | (90.2%) | | 128 | (85.3%) |  |
| Earlier hospitalized/inpatient treatment | 101 | (21.6%) | 55 | (34.2%) | | 15 | (15.8%) | | 6 | (9.8%) | | 25 | (16.7%) |  |
| Psychiatric history family (1^st^ degree) | 208 | (44.4%) | 63 | (39.1%) | | 50 | (52.1%) | | 23 | (37.7%) | | 72 | (48.0%) |  |
| Past Life event | 262 | (56.0%) | 83 | (51.6%) | | 53 | (55.2%) | | 40 | (65.6%) | | 86 | (57.3%) |  |
| Past (complex trauma) | 48 | (10.3%) | 10 | (6.2%) | | 15 | (15.6%) | | 9 | (14.8%) | | 14 | (9.3%) |  |
| Psychotropic medication | 101 | (21.6%) | 33 | (20.4%) | | 22 | (22.7%) | | 11 | (18%) | | 35 | (23%) |  |
| Currently working/studying | 345 | (76.2%) | 111 | (72.5%) | | 77 | (82.2%) | | 45 | (76.3%) | | 112 | (75.7%) |  |
| Problematic financial situation | 45 | (9.5%) | 12 | (7.5%) | | 10 | (10.4%) | | 5 | (8.2%) | | 17 | (11.3%) |  |
